# Supplementary material for: Bombus terrestris Prefer Mixed-Pollen Diets for a Better Colony Performance: A Laboratory Study
Source: Insects. 2024 Apr 17;15(4):285. doi: 10.3390/insects15040285 (PMC11049852; doi:10.3390/insects15040285)
Supplement: Supplementary file 1 [file insects-15-00285-s001.zip › insects-2813841-supplementary.pdf]

Supplementary Materials for

Bombus terrestris Prefer Mixed-Pollen Diets for a Better Colony Performance: A Laboratory Study

Ziyu Zhou, Hong Zhang, Shibonage K. Mashilingi, Chunting Jie, Baodi Guo, Yi Guo, Xiao Hu, Shahid Iqbal, Bingshuai Wei,

Yanjie Liu, and Jiandong An

Corresponding authors: liuyanjie@caas.cn (Y.L.); anjiandong@caas.cn (J.A.)

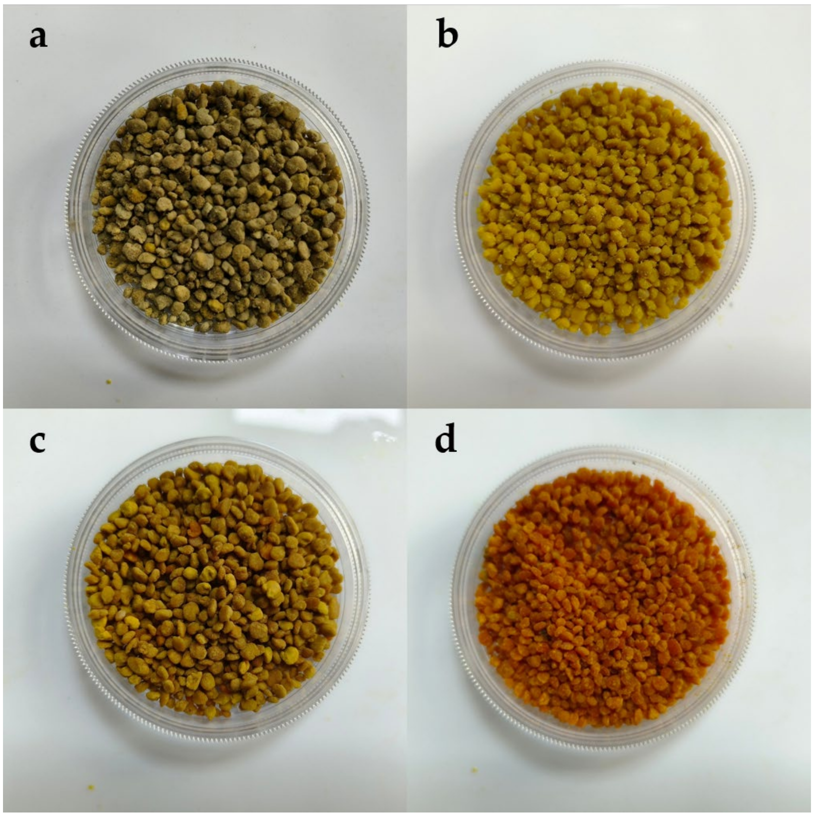

Figure S1. The four pollens used in this study. (a) Wild apricot pollen; (b) oilseed rape pollen; (c) buckwheat pollen; (d) sunflower pollen.

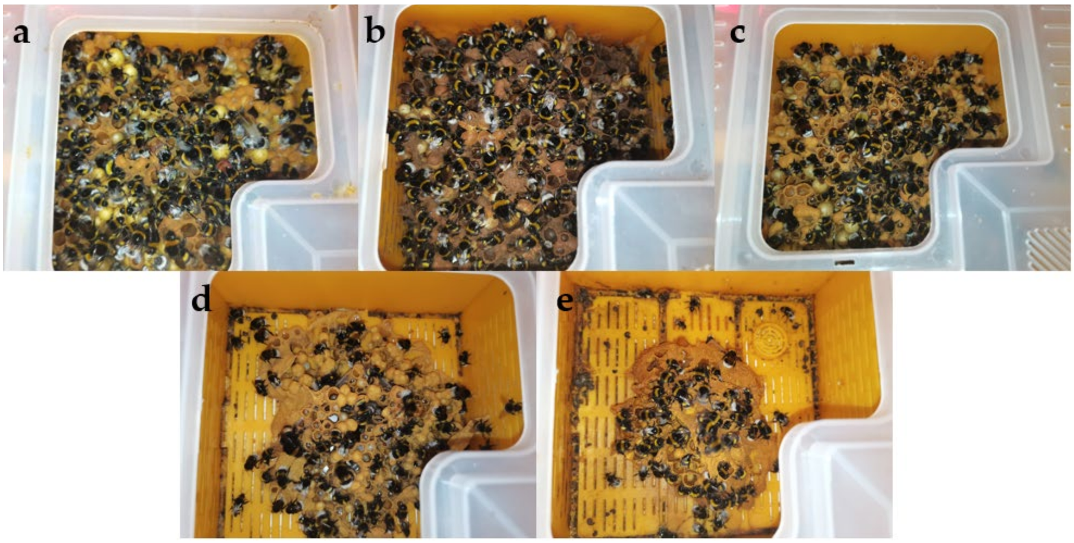

Figure S2. The colony sizes of bumblebees fed the different pollen diets. (a) The mixed four-pollen diet; (b) wild apricot pollen diet; (c) oilseed rape pollen diet; (d) buckwheat pollen diet; (e) sunflower pollen diet.

Table S1. The location sites of the four pollen species collected in this study.

Sites	Wild apricot	Oilseed rape	Buckwheat	Sunflower
1	Yanqing, Beijing	Huangmei, Hubei	Jingbian, Shaanxi	Guyang, Inner Mongolia
2	Miyun, Beijing	Hukou, Jiangxi	Zizhou, Shaanxi	Wuyuan, Inner Mongolia
3	Xinglong, Hebei	Wangjiang, Anhui	Wuyuan, Inner Mongolia	Suide, Shaanxi
